# Spread Through Air Spaces in Multiple Synchronous Bilateral Primary Lung Cancer: Prognostic Implications in a Surgical Cohort

**DOI:** 10.1093/icvts/ivag209

**Published:** 2026-07-25

**Authors:** Stefano Bongiolatti, Simone Tombelli, Antonio Burlone, Lavinia Gatteschi, Giovanni Mugnaini, Rossella Oselin, Ilaria Zenobi, Luca Voltolini

**Affiliations:** Thoracic Surgery Unit, Careggi University Hospital, Florence, 50134, Italy; Thoracic Surgery Unit, Careggi University Hospital, Florence, 50134, Italy; Thoracic Surgery Unit, Careggi University Hospital, Florence, 50134, Italy; Thoracic Surgery Unit, Careggi University Hospital, Florence, 50134, Italy; Thoracic Surgery Unit, Careggi University Hospital, Florence, 50134, Italy; Thoracic Surgery Unit, Careggi University Hospital, Florence, 50134, Italy; Thoracic Surgery Unit, Careggi University Hospital, Florence, 50134, Italy; Thoracic Surgery Unit, Careggi University Hospital, Florence, 50134, Italy; Department of Experimental and Clinical Medicine, University of Florence, Florence, 50134, Italy

**Keywords:** multiple synchronous bilateral primary lung cancer, staged surgery, STAS, survival

## Abstract

**Objectives:**

The surgical treatment of multiple synchronous bilateral primary lung cancer (mSBPLC) showed promising results and the aims of this retrospective study were to assess the oncologic outcomes and the presence of risk factors of worse survival.

**Methods:**

Patients underwent radical (all lesions removed) lung resection for mSBPLC from 2017 to 2024 were included. Exclusion criteria: patients unfit for bilateral surgery, pneumonectomy, multifocal ground-glass opacities, clinical stage IIIA or more, and preoperative treatment. Overall and disease-free survival (DFS) analyses were conducted with the Kaplan-Meier method and log-rank test, Cox-regression analysis was used to identify the predictors of worse survival.

**Results:**

During the study period, 64 patients were screened for the presence of bilateral lung nodules, 45 patients (median age 69 years) were operated for mSBPLC and during the follow-up (median 34 months) we observed 11 deaths and 15 cancer recurrence. In the 80% of patients the main cancer was solid, contralateral was a part-solid in 20%, or pure ground-glass opacity in 20%. Adenocarcinoma was present in 77.8% at first surgery and in 80% at the second. Spread Through the Air Spaces (STAS) was present in 17.8%. The 5-year overall and DFS rate were 77% (median 86 months, 95% CI 72.4-99.5-) and 57% (median 69 months, 95% 32-105.9), respectively. Comparing survivals among patients with and without STAS, we had an overall survival of 0% vs 85% (86 vs 29 months, *P* < .01) and a DFS of 0% vs 63% (69 vs 9 months, *P* < .01). The multivariable analysis demonstrated STAS as significant predictor of worse overall (HR 7.08, *P* = .02) and DFS (HR 5.63, *P* = .017).

**Conclusions:**

Taking into account the small and highly selected study population, staged bilateral surgery could be considered safe and oncologically adequate, showing the association between the presence of STAS and unfavourable long-term outcomes.

## Graphical abstract

**Figure:**
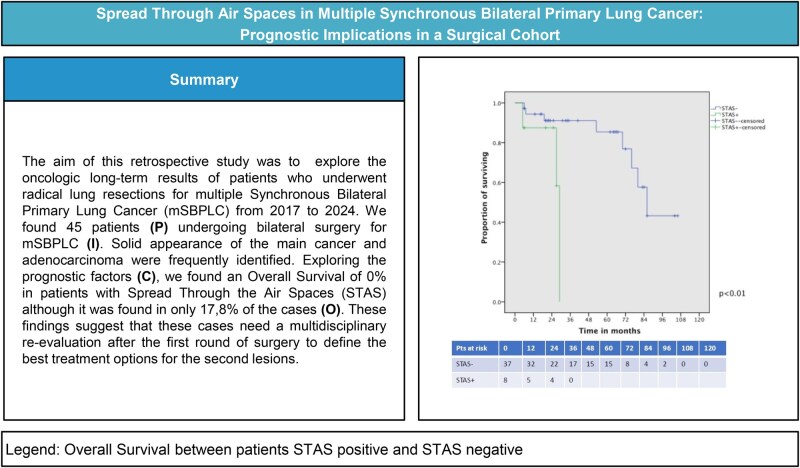

## INTRODUCTION

The increasing implementation of low-dose CT screening programs has significantly improved the detection of early-stage lung cancer. As a consequence, the identification of multiple pulmonary nodules, including bilateral lesions, has become more frequent in clinical practice.^1^ Among these scenarios, multiple synchronous bilateral primary lung cancer (mSBPLC) represents a particularly challenging condition because diagnosis, staging, and treatment differ substantially from those of intrapulmonary metastases (IPM). The distinction between synchronous primary tumours and metastatic disease is crucial. Historically, Martini and Melamed first proposed diagnostic criteria for multiple primary lung cancers in 1975,^2^ which were later refined by the American College of Chest Physicians (ACCP) guidelines and incorporated into modern staging systems.^3^^,^^4^ According to current recommendations, the evaluation of multiple lung nodules should include radiological assessment, histopathological comparison, and, when necessary, immunohistochemical and molecular analyses to determine whether lesions represent independent primaries or metastatic spread.

This distinction has important therapeutic implications. IPM is usually treated with systemic therapies and is associated with poorer long-term outcomes,^5^ whereas mSBPLC can be managed with local treatments, particularly surgery, which remains the gold standard and has been associated with favourable survival rates ranging from 34% to 82%.^6–9^

Several prognostic factors have been investigated in patients with synchronous lung cancers, including lymph node involvement,^10^^,^^11^ tumour size,^10^^,^^12^^,^^13^ biological characteristics,^10^ treatment strategies,^1^ and radiological features on CT imaging.^14^ However, the prognostic role of spread through air spaces (STAS), which has emerged as an important negative prognostic factor in lung cancer,^15^ has not been extensively explored in patients with mSBPLC. The concept of STAS was introduced in the 2015 World Health Organization classification: it is a distinct histopathological pattern of invasion, first described in lung adenocarcinoma, and characterized by the presence of tumour cells, either as small clusters, micropapillary aggregates, solid nests or even single cells, within alveolar air spaces of the lung parenchyma beyond the defined edge of the main tumour mass, without evidence of direct connection to the primary tumour on gross inspection. Multiple studies and a recent meta-analysis have demonstrated that STAS is associated with significantly worse outcomes.^16^ In the context of synchronous multiple primary adenocarcinoma, Qiu et al^17^ recently demonstrated that STAS strongly affected oncologic outcomes, particularly when present in both tumours. Nevertheless, their cohort mainly included ipsilateral lesions, and only a minority of patients presented with bilateral disease. Therefore, the primary aim of the present study was to evaluate the long-term oncologic outcomes of staged bilateral surgery for patients with mSBPLC. Secondary objectives included assessing safety in terms of morbidity and mortality and identifying prognostic factors associated with survival.

## METHODS

We retrospectively reviewed the prospectively maintained database of the Thoracic Surgery Unit at Careggi University Hospital, including all patients who underwent surgery for mSBPLC between January 2017 and December 2025.

Among all the patients who underwent pulmonary resection for NSCLC only the ones with clinical diagnosis of mSBPLC who received bilateral surgical treatment with complete resection (R0) were included in the analysis. Diagnosis of mSBPLC was established according to the Martini and Melamed criteria with contemporary refinements,^2–4^ requiring histologically malignant lesions that were anatomically distinct, without evidence of intrapulmonary metastasis, mediastinal invasion, or extrathoracic disease. In cases of identical histology, lesions were required to be located in different lobes or segments and to show independent biological or molecular characteristics.

Exclusion criteria included functional or anatomical unresectability, non-NSCLC histology, pulmonary metastases from extrapulmonary malignancies, neuroendocrine tumours, bilateral multifocal pure ground-glass opacities, metachronous tumours, unresectable additional lesions, clinical stage IIIA or higher, neoadjuvant treatment, pneumonectomy, and preinvasive lesions such as adenocarcinoma *in situ* or minimally invasive adenocarcinoma.

All patients underwent comprehensive staging including contrast-enhanced CT, PET-CT, and brain MRI when indicated. Invasive mediastinal staging with EBUS/EUS or mediastinoscopy was performed in every case to exclude hilar or mediastinal nodal disease. Due to the long study period (2017-2025) encompassing 2 staging systems, we chose to define all the clinical and pathological staging according to the eighth edition of the AJCC TNM classification. Preoperative functional assessment included complete spirometry, testing the diffusion capacity for carbon monoxide (DLCO), arterial blood gas analysis, cardiopulmonary exercise testing (CPET), and/or lung perfusion scintigraphy when required, as well as cardiac evaluation and routine laboratory tests. Treatment strategies were discussed within an institutional multidisciplinary tumour board (MTB). A staged surgical approach was generally adopted, prioritizing resection of the lesion with the highest clinical stage. Anatomical resection with systematic or lobe-specific lymph node dissection was performed during the first operation, whereas the second procedure was tailored according to residual pulmonary function, favouring parenchyma-sparing resections when oncologically appropriate. Postoperative follow-up included clinical evaluation and contrast-enhanced chest CT every 6 months.

### Statistical analysis

Statistical analysis was performed using IBM SPSS Statistics 24.0 software (SPSS Inc., Chicago, IL). Continuous variables are expressed as median and interquartile range (IQR) and compared with non-parametric test as Mann-Whitney; categorical variables were expressed as count and percentages and compared with chi-square or Fisher’s exact test as indicated. To identify clinical risk factors for the presence of STAS, we constructed univariable and multivariable logistic regression analysis inserting variables with *P* < .2 in the multivariable model. The Kaplan-Meier method was used to estimate overall, disease-free survival (DFS) and inverse Kaplan-Meier method was used to evaluate the median follow-up time. Overall survival (OS) was the time between second operation and death with patients without event being censored at the last follow-up date (31/12/2025); DFS was calculated for those patients who received resection from the date of second surgery to the date of the first evidence of recurrence or death with patients without event being censored at the last follow-up date. Differences in OS and DFS were determined by log-rank analysis. The impact of clinical variables such as age, sex, consolidation-to-tumour ratio, 18-FDG PET-CT avidity, surgical procedures, pathology, pathologic stages, presence of STAS, presence of KRAS mutation, selected *a priori*, on OS and DFS was then evaluated by multivariable Cox-regression model using a significance as *P* < .05.

### Ethical statement

The study was conducted in accordance with the principles of Declaration of Helsinki. This retrospective study was reviewed and approved by the local Ethics Committee (Comitato Etico Regione Toscana—Area Vasta Centro CERT-AVC, approval number 29533_PF, 23/09/2025). In accordance with Italian laws for observational studies, granted a waiver of informed consent from study participants. No collection and storage of data or biological material from research participants were applied in this research.

## RESULTS

During the study period, among 3345 patients who underwent lung resection for NSCLC in our centre, 64 patients were screened for the presence of bilateral lung nodules, but only 45 patients (1.34%) were operated for mSBPLC and during the follow-up (median 34 months) we observed 11 deaths and 15 cancer recurrence (**Figure S1**). Demographical and functional data are resumed in **Table 1**. All surgical variables and pathologic data are resumed in **Table 2**.

**Table 1.** Demographical and Functional Data of the Whole Study Population

Variable *n* = 45	Median/*n*	IQR/%
Median age (IQR)	69	12
Sex female	24	53.30%
Smoke history		
Current	19	42.20%
Former	17	37.80%
Never	9	20%
Main comorbidity		
CAD	3	6.70%
COPD	13	28.90%
Renal failure	1	2.20%
Other cancer	18	40%
Other respiratory	2	4.40%
Autoimmune disease	1	2.20%
FEV1% pre-op	92	25
FVC% pre-op	103	28
DLCO% pre-op	74	21
CT aspect main lesion		
Solid	36	80%
Part-solid	9	20%
Pure-GGO	0	0
CT aspect second lesion		
Solid	27	60%
Part-solid	9	20%
Pure-GGO	9	20%
PET main lesion		
Intense	27	84.40%
Moderate	3	6.70%
Mild	6	13.30%
No uptake	2	4.40%
PET second lesion		
No uptake	10	22.20%
Mild	8	17.80%
Moderate	6	13.30%
Intense	13	82.20%
FEV1% second	87	27
FVC% second	96	22
DLCO% second	67	25
Median delta FEV1%	−6.39	15.46
Median delta FVC%	−6.44	21.36
Median delta DLCO%	−4.82	13.71
Median delta FVC%	−6.44	21.36

Abbreviations: CAD, coronary artery disease; COPD, chronic obstructive pulmonary disease; DLCO, diffusing capacity of the lungs for carbon monoxide; FEV1, forced expiratory volume at the first second; FVC, forced vital capacity; GGO, ground glass opacity; IQR, interquartile range; PET, positron emission tomography.

**Table 2.** Surgical Data of the Whole Study Population

Variable *n* = 45	Median/*n*	IQR/%
First surgery		
RUL	8	17.8%
LUL	10	22.2%
ML	1	2.2%
RLL	6	13.3%
Single segment	11	24.4%
Multiple segments	2	4.4%
Wedge resection	7	15.6%
Lymph node assessment first		
Systematic	29	64.4%
Lobe specific	10	22.2%
Sampling	5	11.1%
No	1	2.2%
Minimally invasive surgery first	39	86.7%
Complications first	11	24.4%
CDI first		
1	1	2.2%
2	8	17.8%
3a (bronchoscopy)	1	2.2%
4a (stroke)	1	2.2%
Median maximum diameter	21	13
Second surgery		
RUL	1	2.2%
LUL	2	4.4%
ML	1	2.2%
RLL	1	2.2%
Single segment	13	28.9%
Multiple segments	3	6.7%
Wedge resection	24	53.3%
Minimally invasive surgery second	37	82.2%
Complications second	7	15.6%
CDI second		
1	1	2.2%
2	5	11.1%
3b (PAL with need of surgery)	1	2.2%
Lymph node assessment second		
Systematic	14	31.1%
Lobe specific	14	31.1%
Sampling	11	24.4%
No	6	13.3%
Median maximum diameter	16	11
Recurrence	15	33.3%
Site		
Local	7	15.6%
Regional	5	11.1%
Distant	7	15.6%
Adjuvant treatment	7	15.6%

Abbreviations: CDI, Clavien-Dindo index; IQR, interquartile range; LUL, left upper lobectomy; ML, middle lobectomy; PAL, prolonged air leak; RLL, right lower lobectomy; RUL, right upper lobectomy.

### First stage

5The larger part of patients had a solid main cancer (80%), demonstrating high 18 FDG avidity at the PET-CT-scan (84.4%) and consequently underwent lobectomy (*n* = 25/45, 55%) with systematic or lobe-specific lymph node dissection in 86.7% with a minimally invasive approach in 86.7%. No 30-day or 90-day mortality was observed and the complication rate was 24.4% (almost CDI 2). Pathology revealed ADC in the larger part of patients (77.8%) with predominant acinar or papillary morphology, whereas 26.7% of patients had high-grade ADC pattern (HG-ADC). Stage I was observed in 33 patients (82.5%) and STAS was present in 7 (15.6%), whereas KRAS and EGFR mutation in 20% and 8.9%, respectively (**Table 3**).

**Table 3.** Comparison Between First and Second Specimen Pathological and Biological Data

Variable main lesion *n* = 45	Median/*n*	IQR/%	Variable second lesion *n* = 45	Median/*n*	IQR/%
Pathology main lesion			Pathology second lesion		
ADC	35	77.80%	ADC	36	80%
SCC	9	20%	SCC	8	17.80%
Other	1	2.20%	Other	1	2.20%
ADC subtype			ADC subtype		
Solid	3	6.70%	Solid	4	8.90%
Micropapillar	1	2.20%	Micropapillar	1	2.20%
Papillar	12	26.70%	Papillar	12	26.70%
Acinar	14	31.10%	Acinar	8	17.80%
Lepidic	2	4.40%	Lepidic	2	4.40%
Mucinous	3	6.70%	Other	8	17.80%
ADC second component			ADC second		
Solid	2	4.40%	Solid	3	6.70%
Micropapillar	4	8.90%	Micropapillar	3	6.70%
Papillar	3	6.70%	Papillar	5	11.10%
Acinar	4	8.90%	Acinar	6	13.30%
Lepidic	4	8.90%	Other	1	2.20%
Other	1	2.20%			
HG-ADC	12	26.70%	HG-ADC second	19	42.20%
P stage first			P stage second		
1a1	6	13.30%	1a1	7	15.65
1a2	11	24.40%	1a2	19	42.20%
1a3	11	24.40%	1a3	9	20%
1b	5	11.10%	1b	1	2.20%
2a	3	6.70%	2a	1	2.20%
2b	6	13.30%	2b	4	8.90%
3a	3	6.70%	3a	3	6.70%
			3b	1	2.20%
R0 resection	45	100%	R0 resection	37	82.20%
			R0 uncertain	8	17.80%
EGFR first	4	8.90%	EGFR second	3	6.70%
KRAS first	9	20%	KRAS second	13	28.90%
STAS first	7	15.60%	STAS second	2	4.40%

Abbreviations: ADC, adenocarcinoma; EGFR, epidermal grow factor receptor; HG-ADC, high-grade adenocarcinoma; IQR, interquartile range; KRAS, Kirsten rat sarcoma; MIP, micropapillary; SCC, squamous cell carcinoma; STAS, spread through the air spaces.

### Second stage

The second staged surgery was performed within 1 year after complete re-staging and functional assessment in 22 patients. Data are shown in **Table 3**. The parenchymal sparing strategy was largely applied for the second surgery and sublobar resections were performed in 40 patients (88.8%) with lymph node assessment in 86.7%. Again, no 30-day nor 90-day mortality was observed and the complication rate was 15.6% (almost CDI 2). Adenocarcinoma and stage I were the most frequent finding (80% each); KRAS, STAS, and EGFR were identified in 28.9%, 4.4%, and 6.7% of patients, respectively. The presence of STAS was unilateral in 7 cases (15.6%) and bilateral in 1 (2.2%); KRAS was unilaterally distributed in 14 (31.1%) and bilaterally in 4 (8,8%) patients (**Table 3**).

### Oncologic long-term results

**Figure 1.**
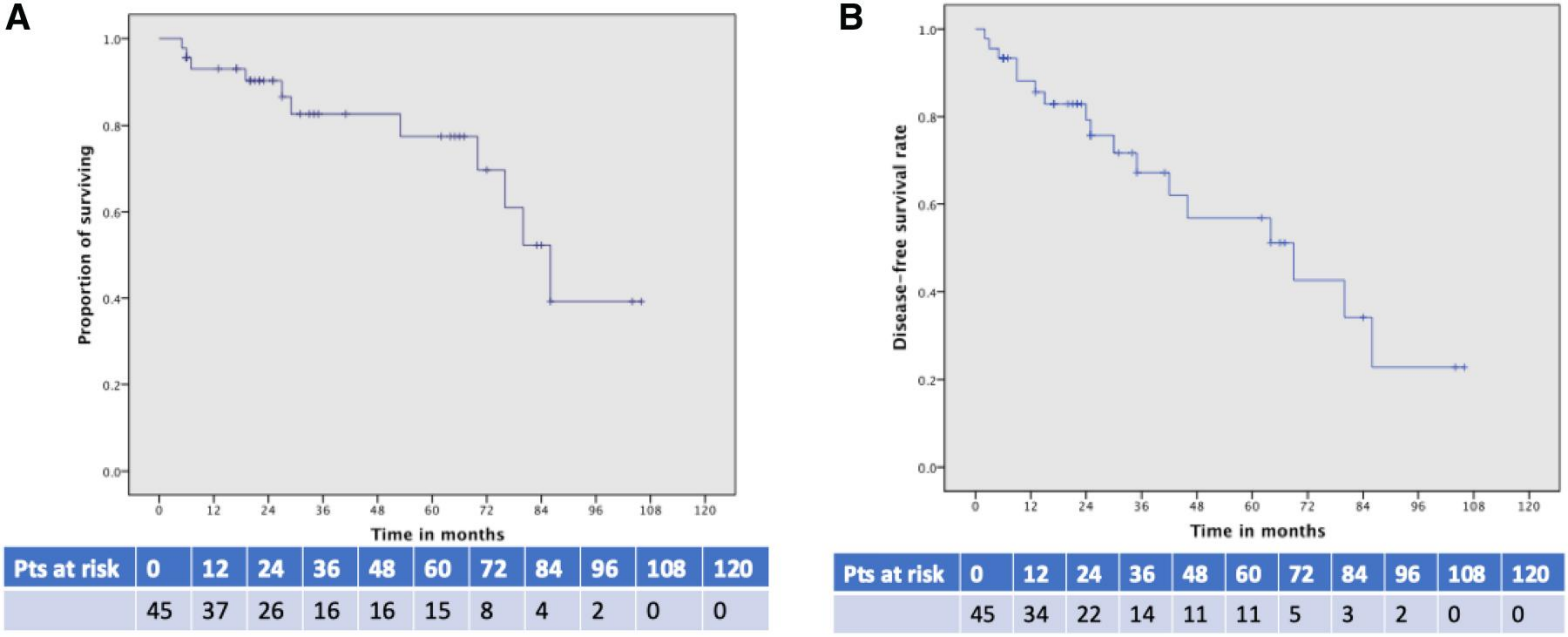
Overall (A) and disease-free survival (B) curves of the whole study population

**Figure 2.**
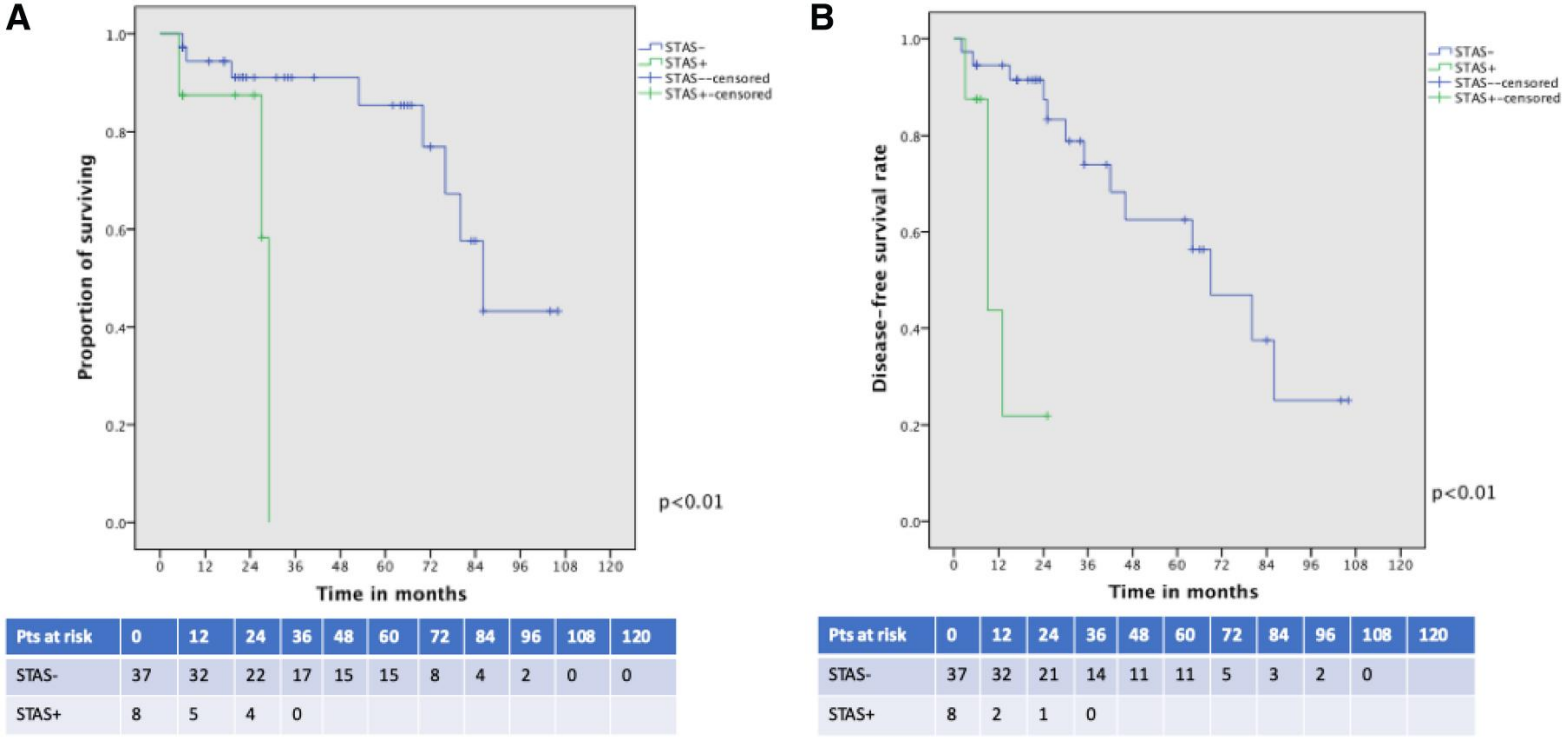
Overall (A) and disease-free survival (B) curves comparing patients with the presence of STAS and without STAS

At the median follow-up of 34 months (95% CI 17.9-50), 11 patients (24.4%) have died: 9 for cancer relapse, 1 for complications of long-term cardiac disease, and 1 for sequalae of peripheral vascular disease associated with renal failure. The estimated median OS was 86 months (95% CI 72.4-99.5) and the 5-year OS was 77% for the whole cohort (**Figure 1A**). During the follow-up period, *n* = 15 (33.3%) patients developed cancer recurrence: 7 local, 5 regional, and 7 distant (2 patients had local and distant and one patient had local, regional, and distant recurrences). The 5-year DFS was 57% and the median DFS was not reached within 5 years (estimated median DFS was 69 months, 95% CI 32-105.9) (**Figure 1B**). Exploring the potential risk factors of reduced survivals, we noted significant differences between the OS curves in patients with STAS (86 months, 95% CI 71.1-95.2 vs 29 months, 95% CI 18.7-32, *P* < .01, **Figure 2A**) and in patients with KRAS mutation (86 months, 95% CI 72.1-98.4 vs 70 months, 95% CI 37.1-72, *P* = .02, **Figure S2A**). The analysis of the DFS curves confirmed the significant differences between STAS − and STAS + patients (median 69 months—47.8-90.1—vs 9 months—2.91-15.08, *P* < .01, **Figure 2B**), but not for KRAS + patients (*P* = .19, **Figure S2B**). The Cox-regression analysis indicated that STAS was the only significant risk factor for decreased OS and DFS at the univariable and multivariable analysis (HR 7.08, 95% CI 1.36-36.6, *P* = .02; HR 5.63, 95% CI 1.35-23.4, *P* = .017). Unfortunately, no other risk factors were identified even if the KRAS mutation was associated with a trend towards significance for decreased OS (HR 4.52, 95% CI 0.78-26.07, *P* = .09).

### Analysis on STAS

Following the oncologic long-term results, we divided and compared the whole cohort in 2 groups based on the presence of STAS (**Table S1**). Age (*P* = .019) and adenocarcinoma patterns (*P* = .025 and *P* = .038) at the second resection were significantly different among these groups. The univariable and multivariable regression analysis to identify the risk factors for the presence of STAS showed that only age was the strongest risk factor for the presence of STAS (OR 0.86, 95% CI 0.7-0.986, *P* = .033).

### Additional analysis

Additional survival analyses were performed to explore the combined prognostic impact of STAS and KRAS mutation status. Patients were stratified into 4 groups according to STAS and KRAS expression, with particular focus on double-negative (DN) patients (STAS−/KRAS−) and double-positive (DP) patients (STAS+/KRAS+). A clear survival advantage was observed in DN patients (**Figure S3A and B**). Extended survival analyses were performed to evaluate the impact of the extent of the first resection combined with the presence of STAS: dividing the study population in 2 groups based on performing sublobar resection or lobectomy, we did not report a significant survival advantage for lobectomy in both OS (**Figure S4A**) and DFS (**Figure S4B**).

## DISCUSSION

Multiple synchronous bilateral primary lung cancer represents an uncommon clinical entity but poses significant diagnostic and therapeutic challenges. Correctly distinguishing mSBPLC from IPM is essential, as treatment strategies and prognosis differ substantially. While metastatic disease is typically managed with systemic therapies, true multiple primary tumours may benefit from local treatment, particularly surgery. Previous studies have reported 5-year OS rates ranging from 34% to 56% after surgical treatment of synchronous lung cancers,^1^^,^^6^^,^^10^^,^^11^^,^^18^^,^^19^ with even better outcomes reported in selected patients with bilateral disease as demonstrated by Zhang *et al* with a 82.7% 5-year OS.^7^ In the present study, the estimated 5-year OS and DFS were 77% and 57%, respectively, confirming the potential effectiveness of a surgical strategy in carefully selected patients.

Our results indicate that STAS could represent a prognostic factor affecting survival. Patients with STAS had significantly worse overall and DFS, and STAS remained the only independent predictor of poor outcomes in multivariable analysis. Although KRAS mutation was associated with worse OS, its impact was less pronounced and did not reach statistical significance in the multivariable model. Several prognostic variables previously described in the literature, including lymph node involvement,^10^^,^^11^ tumour size and biology,^10^^,^^12^^,^^13^ and radiological patterns,^14^ were not confirmed in our cohort. This discrepancy may be explained by the relatively small and homogeneous population and by the rigorous preoperative staging protocol, which included systematic PET-CT-scan and invasive mediastinal staging, resulting in a very low rate of pathologic nodal involvement. Radiological characteristics have also been associated with prognosis in synchronous lung cancer. Some study showed favourable long-term results when the second tumour had a ground-glass component.^7^^,^^13^^,^^14^ In our series, however, the limited number of ground-glass lesions prevented the demonstration of a significant association between CT morphology and histologic subtype and survival.

The prognostic relevance of STAS in synchronous lung cancer has recently been highlighted by Qiu et al,^17^ who reported worse outcomes when STAS was present in both tumours. Our findings further support the importance of this pathological feature. STAS is increasingly recognized as a marker of aggressive tumour behaviour and may influence recurrence patterns after lung cancer surgery,^20^^,^^21^ but the therapeutic role of the extent of resection is still a matter of debate: some study showed a prognostic advantage in STAS positive patients undergoing lobectomy, but others did not demonstrate this survival advantage and then we can only assume STAS as a prognostic factors without any strong therapeutic evidences.^22^^,^^23^ Probably in the next future STAS may be included in the staging system and influence therapeutic decision-making, potentially identifying^24^ patients who could benefit from multimodal treatment strategies rather than surgery alone.

Finally, our data confirm the safety of staged bilateral surgery in carefully selected patients. No 30- or 90-day mortality was observed, and postoperative complications were limited. The extensive use of minimally invasive techniques and parenchyma-sparing resections likely contributed to the preservation of postoperative pulmonary function. Brandt et al^1^ demonstrated that staged surgery was the best treatment option using a large administration database. Patients who underwent to SABR bilaterally had significantly lower OS and DFS than patients treated with surgery or surgery plus SABR. Regarding the surgical approach in term of extent of resection, several papers corroborate our approach showing that sublobar resection as second step had low complication profile and safety than bilateral lobectomy not affecting the oncologic long-term results.^19^^,^^25^

This study has several limitations. First, its retrospective single-centre design with strict inclusion and exclusion criteria may introduce selection bias, limit robustness of our results, reduce the statistical power of this analysis and then our results should be considered hypothesis generating and warrant confirmation in larger multicentre cohorts. Second, immunohistochemical and molecular data were not available for all patients, limiting additional exploratory analyses. Third, the small sample size (study population of 45 patients) and the low number of events (11 deaths and 15 recurrences) reduced the statistical power of regression models. Moreover, the incidence of STAS in our cohort was lower than that reported in Eastern series, which may further limit the generalizability of our findings. Although the follow-up period could be considered short for long-term survival analyses, our results showed that STAS increased the risk of early cancer recurrence and poor survival outcomes few months after resection. Finally, the absence of a comparison cohort treated with alternative strategies such as SABR or combined treatments prevents direct evaluation of different therapeutic approaches. Regarding the possible predictors of STAS, we cannot use the SUVmax because it was not reported in all the PET-CT-scan available so we used a non-numeric scale with intrinsic limits.

## CONCLUSIONS

Our study demonstrates that staged and parenchyma-sparing bilateral surgery represents a safe and effective treatment strategy for patients with mSBPLC when performed after careful functional evaluation and accurate clinical staging. Although in a limited study population, the long-term oncologic outcomes may be influenced by tumour biology, suggesting STAS as a potential prognostic factor associated with poor survival. Large multicentre studies are the only way to determine the actual impact of STAS and its interaction with the extent of resection.

## Supplementary Material

ivag209_Supplementary_Data

## Data Availability

The data underlying this article will be shared on reasonable request to the corresponding authors.

## ABBREVIATIONS

ACCP: American College of Chest Physicians
ADC: adenocarcinoma
CDI: Clavien-Dindo index
CPET: cardiopulmonary exercise testing
DFS: disease-free survival
DLCO: diffusion capacity for carbon monoxide
DN: double-negative
HG-ADC: high-grade ADC pattern
IPM: intrapulmonary metastases
mSBPLC: multiple synchronous bilateral primary lung cancer
MTB: multidisciplinary tumour board
NSCLC: non-small cell lung cancer
OS: overall survival
PET-CT: positron emission tomography/CT
STAS: spread through the air spaces
