# Supplementary material for: Spread Through Air Spaces in Multiple Synchronous Bilateral Primary Lung Cancer: Prognostic Implications in a Surgical Cohort
Source: Interdiscip Cardiovasc Thorac Surg. 2026 Jul 25;41(8):ivag209. doi: 10.1093/icvts/ivag209 (PMC13453306; doi:10.1093/icvts/ivag209)
Supplement: Supplementary file 1 [file ivag209_supplementary_data.zip › supplemental mat 1.docx]

| Variable | STAS neg (n=37) | STAS pos (n=8) | p |
| --- | --- | --- | --- |
| Males | 20 (54.1%) | 4 (50%) | 1 |
| Current smoker  Former  Never | 15 (40.5%)  14 (37.8%)  8 (21.6%) | 4 (50%)  3 (37.5%)  1 (12.5%) | 0.81 |
| CT 1st cancer  Solid  Part solid | 29 (78.4%)  8 (21.6%) | 7 (87.5%)  1 (12.5%) | 0.55 |
| CT second cancer  Solid  Part-solid  Pure-GGO | 22 (59.5%)  8 (21.6%)  7 (18.9%) | 5 (62.5%)  1 (12.5%)  2 (25%) | 0.81 |
| Sublobar resection 1^st^ | 16 (43.2%) | 4 (50%) | 0.72 |
| Sublobar resection 2^nd^ | 32 (86.5%) | 8 (100%) | 0.27 |
| Both sublobar resection | 11 (29.7%) | 4 (50%) | 0.27 |
| Intense FDG PET-CT avidity 1^st^ | 22 (73.3%) | 5 (62.5%) | 0.21 |
| Intense FDG PET-CT avidity 2^nd^ | 11 (36.7%) | 2 (28.6%) | 0.16 |
| Pathology 1^st^  ADC  SCC  other | 28 (75.7%)  8 (21.6%)  1 (2.7%) | 7 (87.5%)  1 (12.5%) | 0.73 |
| Predominant ADC subtype 1^st^  Solid  MIP  Papillary  Acinar  Lepidic  other | 2 (7.1%)  1 (3.6%)  9 (32.1%)  12 (42.9%)  1 (3.6%)  3 (10.7) | 1 (14.3%)  0  3 (42.9%)  2 (28.6%)  1 (14.3%)  0 | 0.71 |
| Second component ADC 1^st^  Solid  MIP  Papillary  Acinar  Lepidic  other | 1 (7.7%)  2 (15.4%)  1 (7.7%)  4 (30.8%)  4 (30.8%)  1 (7.7%) | 1 (20%)  2 (40%)  2 (40%)  0  0  0 | 0.2 |
| HG-ADC 1^st^ | 8 (28.6%) | 4 (57.1%) | 0.2 |
| P stage 1^st^  1a1  1a2  1a3  1b  2a  2b  3a | 6 (16.2%)  8 (21.6%)  9 (24.3%)  4 (10.8%)  2 (5.4%)  5 (13.5%)  3 (8.1%) | 0  3 (37.5%)  2 (25%)  1 (12.5%)  1 (12.5%)  1 (12.5%)  0 | 0.79 |
| P stage 2^nd^  1a1  1a2  1a3  1b  2a  2b  3a  3b | 7 (18.9%)  13 (35.1%)  9 (24.3%)  1 (2.7%)  1 (2.7%)  4 (10.8%)  1 (2.7%)  1 (2.7%) | 0  6 (75%)  0  0  0  0  2 (25%)  0 | 0.089 |
| Pathology 2^nd^  ADC  SCC  other | 28 (75.7%)  8 (21.6%)  1 (2.7%) | 8 (100%)  0  0 | 0.29 |
| Predominant ADC subtype 2^nd^  Solid  MIP  Papillary  Acinar  Lepidic  other | 2 (7.4%)  0  11 (40.7%)  4 (14.8%)  2 (7.4%)  0 | 2 (25%)  1 (12.5%)  1 (12.5%)  4 (50%)  0  0 | 0.025 |
| Second component ADC 2^nd^  Solid  MIP  Papillary  Acinar  other | 2 (18.2%)  0  2 (18.2%)  6 (54.5%)  1 (9.1%) | 1 (14.3%)  3 (42.9%)  3 (42.9%)  0  0 | 0.038 |
| HG-ADC 2nd | 14 (53.8%) | 5 (62.5%) | 0.66 |
| Median diameter 1^st^ | 21 (15) | 21.5 (17) | 1 |
| Median diameter 2^nd^ | 16 (11) | 15 (5) | 0.33 |
| Median age | 71 (9) | 61 (9) | 0.019 |
| Recurrence | 11 (29.7%) | 4 (50%) | 0.27 |
| Local recurrence | 5 (45.5%) | 2 (50%) | 0.87 |
| Regional recurrence | 5 (45.5%) | 0 | 0.099 |
| Distant recurrence | 4 (36.4%) | 3 (75%) | 0.18 |
| Adjuvant therapy | 6 (23.1%) | 1 (14.3%) | 0.61 |

Table 4. Comparison between STAS positive and STAS negative clinical data (STAS: spread trough the air apaces; CT: computed tomography; GGO: ground glass opacity; PET: Positron Emission Tomography; ADC: adenocarcinoma; SCC: squamous cell carcinoma; MIP: micropapillary; HG-ADC: high-grade adenocarcinoma).
