# Supplementary material for: Spread Through Air Spaces in Multiple Synchronous Bilateral Primary Lung Cancer: Prognostic Implications in a Surgical Cohort
Source: Interdiscip Cardiovasc Thorac Surg. 2026 Jul 25;41(8):ivag209. doi: 10.1093/icvts/ivag209 (PMC13453306; doi:10.1093/icvts/ivag209)
Supplement: Supplementary file 1 [file ivag209_supplementary_data.zip › figure 2ab suppl.pdf]

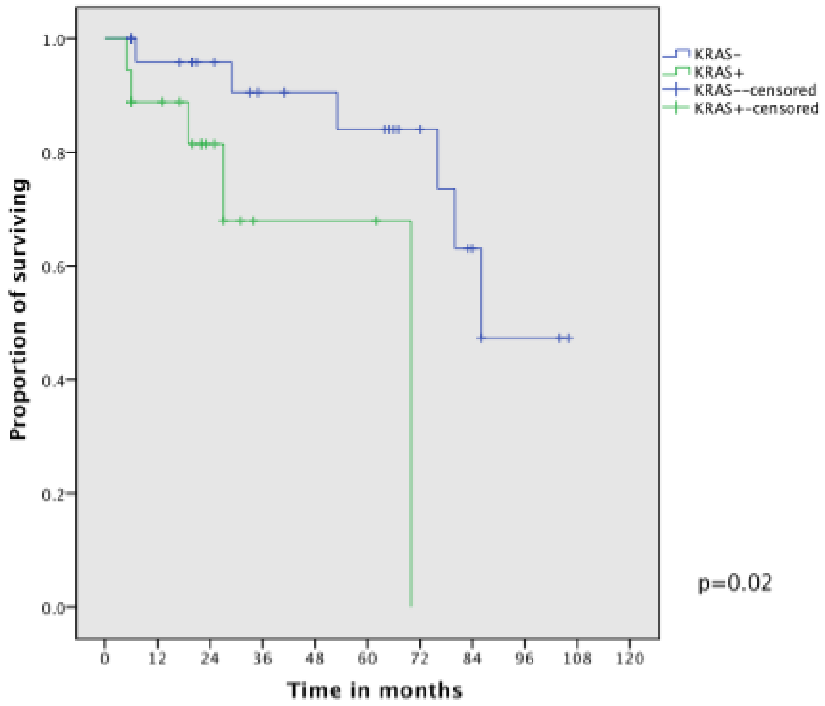


| Pts at risk | 0 | 12 | 24 | 36 | 48 | 60 | 72 | 84 | 96 | 108 | 120 |
|---|---|---|---|---|---|---|---|---|---|---|---|
| KRAS- | 27 | 23 | 19 | 15 | 14 | 13 | 8 | 4 | 2 | 0 | 0 |
| KRAS+ | 18 | 14 | 7 | 2 | 2 | 2 | 0 | | | | |

A

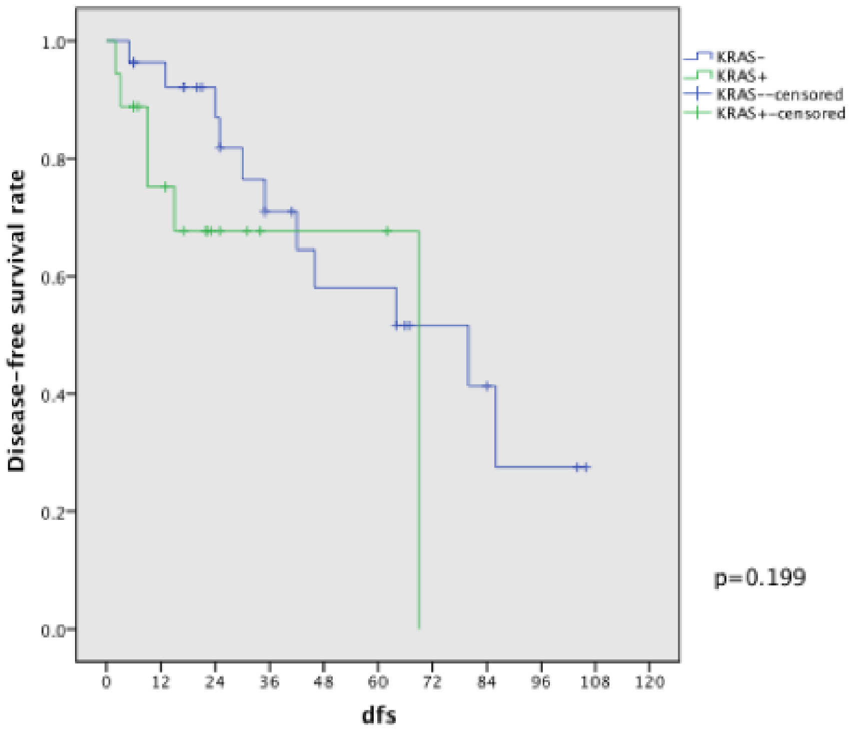


| Pts at risk | 0 | 12 | 24 | 36 | 48 | 60 | 72 | 84 | 96 | 108 | 120 |
|---|---|---|---|---|---|---|---|---|---|---|---|
| KRAS- | 27 | 23 | 17 | 12 | 9 | 9 | 5 | 3 | 2 | 0 | |
| KRAS+ | 18 | 11 | 5 | 2 | 2 | 2 | 0 | | | | |

B
