# Supplementary material for: Spread Through Air Spaces in Multiple Synchronous Bilateral Primary Lung Cancer: Prognostic Implications in a Surgical Cohort
Source: Interdiscip Cardiovasc Thorac Surg. 2026 Jul 25;41(8):ivag209. doi: 10.1093/icvts/ivag209 (PMC13453306; doi:10.1093/icvts/ivag209)
Supplement: Supplementary file 1 [file ivag209_supplementary_data.zip › figure 4ab suppl.pdf]

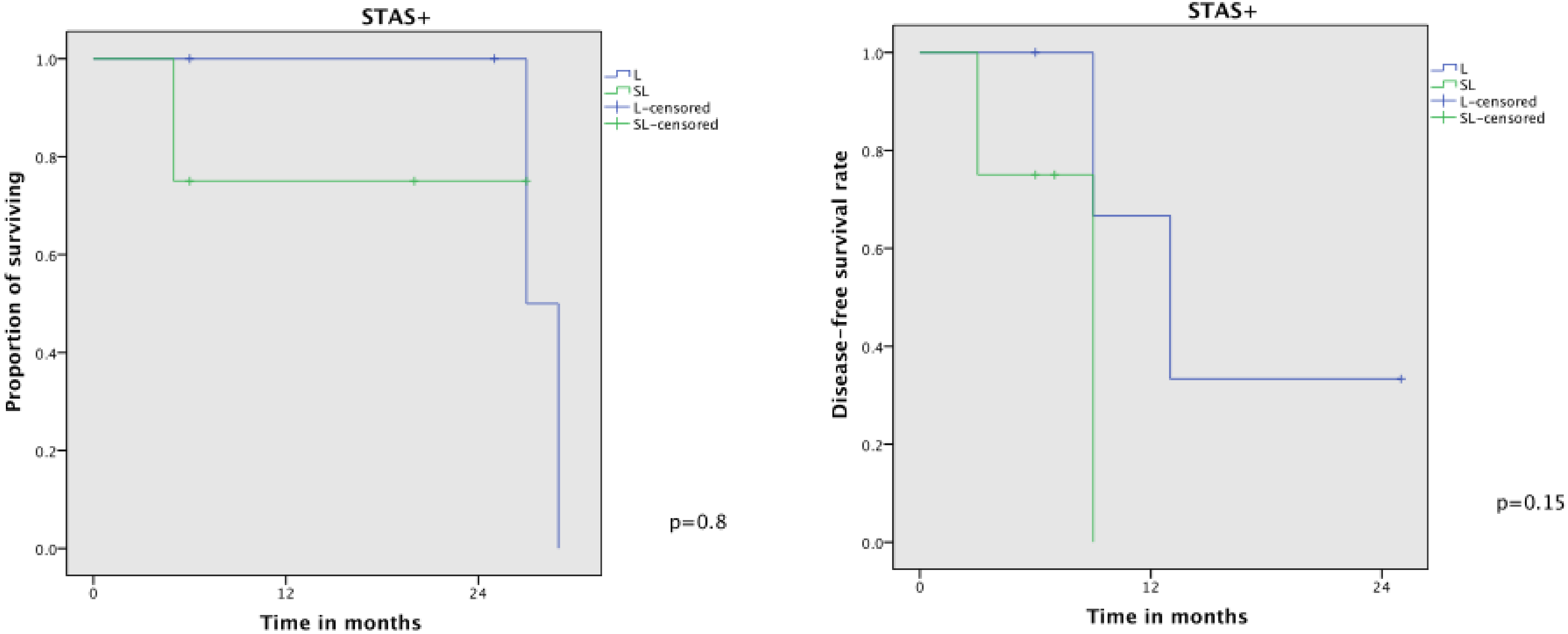
STAS+
Proportion of surviving
1.0
0.8
0.6
0.4
0.2
0.0
0
12
24
Time in months
L
SL
L-censored
SL-censored
p=0.8
STAS+
Disease-free survival rate
1.0
0.8
0.6
0.4
0.2
0.0
0
12
24
Time in months
L
SL
L-censored
SL-censored
p=0.15

A

B
