# Supplementary material for: Spread Through Air Spaces in Multiple Synchronous Bilateral Primary Lung Cancer: Prognostic Implications in a Surgical Cohort
Source: Interdiscip Cardiovasc Thorac Surg. 2026 Jul 25;41(8):ivag209. doi: 10.1093/icvts/ivag209 (PMC13453306; doi:10.1093/icvts/ivag209)
Supplement: Supplementary file 1 [file ivag209_supplementary_data.zip › figure 1 suppl.pdf]

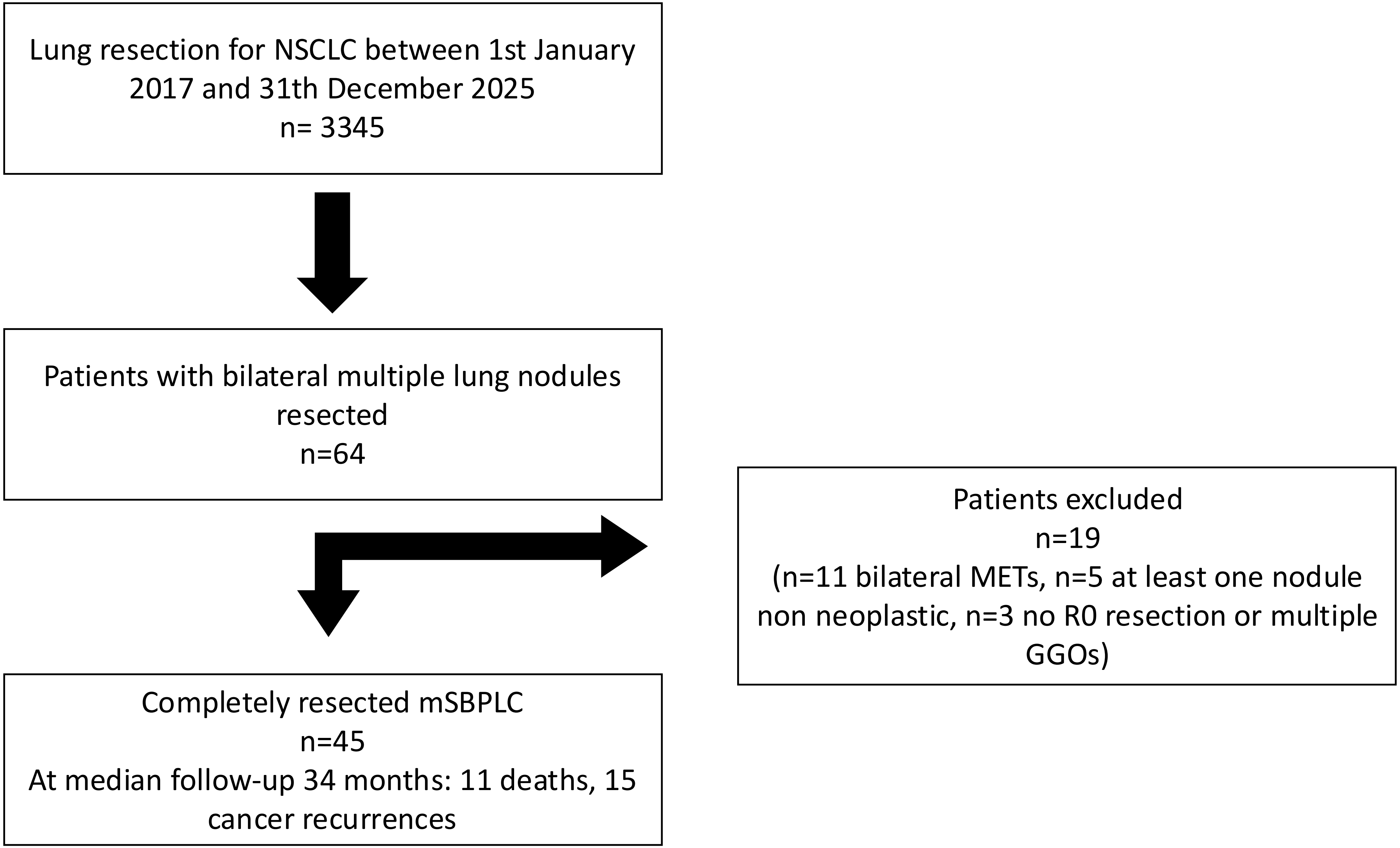
Lung resection for NSCLC between 1st January 2017 and 31th December 2025
n= 3345
Patients with bilateral multiple lung nodules resected
n=64
Patients excluded
n=19
(n=11 bilateral METs, n=5 at least one nodule non neoplastic, n=3 no R0 resection or multiple GGOs)
Completely resected mSBPLC
n=45
At median follow-up 34 months: 11 deaths, 15 cancer recurrences
