# Supplementary material for: Spread Through Air Spaces in Multiple Synchronous Bilateral Primary Lung Cancer: Prognostic Implications in a Surgical Cohort
Source: Interdiscip Cardiovasc Thorac Surg. 2026 Jul 25;41(8):ivag209. doi: 10.1093/icvts/ivag209 (PMC13453306; doi:10.1093/icvts/ivag209)
Supplement: Supplementary file 1 [file ivag209_supplementary_data.zip › SUPPLEMENTARY MATERIAL LIST OF FIGURES.docx]

SUPPLEMENTARY MATERIAL LIST OF FIGURES

Figure 1 supplemental material. Flow chart of the study population selection criteria.

Figure 2 supplemental material. Overall (A) and disease-free survival (B) curves comparing patients with the presence of KRAS mutation and without KRAS mutation.

Figure 3 supplemental material. Overall survival (A) and disease-free survival (B) curves according the subgroup of analysis with the combination of STAS and KRAS status.

Figure 4 supplemental material. Overall (A) and disease-free survival (B) curves comparing STAS+ patients undergoing lobectomy or sublobar resection as first surgical procedure.
